# Mitochondrial Functions, Energy Metabolism and Protein Glycosylation are Interconnected Processes Mediating Resistance to Bortezomib in Multiple Myeloma Cells

**DOI:** 10.3390/biom10050696

**Published:** 2020-04-30

**Authors:** Daniele Tibullo, Cesarina Giallongo, Alessandra Romano, Nunzio Vicario, Alessandro Barbato, Fabrizio Puglisi, Rosalba Parenti, Angela Maria Amorini, Miriam Wissam Saab, Barbara Tavazzi, Renata Mangione, Maria Violetta Brundo, Giacomo Lazzarino, Giuseppe Alberto Palumbo, Giovanni Li Volti, Francesco Di Raimondo, Giuseppe Lazzarino

**Affiliations:** 1 Section of Biochemistry, Department of Biomedical and Biotechnological Sciences, University of Catania, 95123 Catania, Italy; d.tibullo@unict.it (D.T.); amorini@unict.it (A.M.A.); mirisaab@gmail.com (M.W.S.); lazzarig@unict.it (G.L.); 2 Section of Haematology, Department of Medical and Surgical Sciences and Advanced Technologies “G.F. Ingrassia”, University of Catania, 95123 Catania, Italy; cesarinagiallongo@yahoo.it (C.G.); palumbo.ga@gmail.com (G.A.P.); 3 Division of Hematology, Department of General Surgery and Medical-Surgical Specialties, A.O.U. “Policlinico-Vittorio Emanuele”, University of Catania, 95123 Catania, Italy; sandrina.romano@gmail.com (A.R.); alessandrobarbato93@libero.it (A.B.); puglisi.fabri@gmail.com (F.P.); diraimon@unict.it (F.D.R.); 4 Section of Physiology, Department of Biomedical and Biotechnological Sciences, University of Catania, 95123 Catania, Italy; nunziovicario@unict.it (N.V.); parenti@unict.it (R.P.); 5 Institute of Biochemistry and Clinical Biochemistry, Catholic University of Rome, and Fondazione Policlinico Universitario A. Gemelli IRCCS, 00168 Rome, Italy; barabara.tavazzi@unicatt.it (B.T.); renatamangione@hotmail.it (R.M.); 6 Department of Biological, Geological and Environmental Science, University of Catania, 95123 Catania, Italy; mvbrundo@unict.it; 7 UniCamillus-Saint Camillus International University of Health Sciences, Via di Sant’Alessandro 8, 00131 Rome, Italy; giacomo.lazzarino@unicamillus.org

**Keywords:** bortezomib, multiple myeloma, oxidative stress, metabolism, hexosamine biosynthetic pathway

## Abstract

The proteasome inhibitor bortezomib (BTZ) has emerged as an effective drug for the treatment of multiple myeloma even though many patients relapse from BTZ therapy. The present study investigated the metabolic pathways underlying the acquisition of bortezomib resistance in multiple myeloma. We used two different clones of multiple myeloma cell lines exhibiting different sensitivities to BTZ (U266 and U266-R) and compared them in terms of metabolic profile, mitochondrial fitness and redox balance homeostasis capacity. Our results showed that the BTZ-resistant clone (U266-R) presented increased glycosylated UDP-derivatives when compared to BTZ-sensitive cells (U266), thus also suggesting higher activities of the hexosamine biosynthetic pathway (HBP), regulating not only protein *O*- and *N*-glycosylation but also mitochondrial functions. Notably, U266-R displayed increased mitochondrial biogenesis and mitochondrial dynamics associated with stronger antioxidant defenses. Furthermore, U266-R maintained a significantly higher concentration of substrates for protein glycosylation when compared to U266, particularly for UDP-GlcNac, thus further suggesting the importance of glycosylation in the BTZ pharmacological response. Moreover, BTZ-treated U266-R showed significantly higher ATP/ADP ratios and levels of ECP and also exhibited increased mitochondrial fitness and antioxidant response. In conclusions, our findings suggest that the HBP may play a major role in mitochondrial fitness, driving BTZ resistance in multiple myeloma and thus representing a possible target for new drug development for BTZ-resistant patients.

## 1. Introduction

The ubiquitin-proteasome pathway plays a key role in protein processing and degradation, transcriptional regulation, cellular stress responses and antigen presentation [1,2]. Proteasome inhibition causes an imbalance between the production and degradation of proteins, leading to the accumulation of unfolded proteins and endoplasmic reticulum (ER) stress, with the activation of the unfolded protein response (UPR). These mechanisms lead to the dysregulation of Ca^2+^ homeostasis and the activation of cell apoptosis [3,4,5]. Proteasome inhibitors (PI) are extensively used for the therapy of multiple myeloma (MM) and mantle cell lymphoma. Myeloma plasma cells are particularly sensitive to increased ER stress because they constitutively express ER stress survival factors for proper antibody assembly and secretion and, therefore, to function as secretory cells [6]. Thus, MM cells are more susceptible to the cytotoxic effects of PI compared to non-tumoral cells. Among PI, bortezomib (BTZ) is successfully used to treat newly diagnosed and relapsed MM patients, but its efficacy is reduced by the occurrence of drug resistance [7]. The evidence for such is associated with impaired binding of the drug to proteasome subunits, the up-regulation of the proteasomal machinery and an increase in the ratio of BTZ-bound to BTZ-free proteasomal subunits, as a form of acquired proteasomal subunit immunity [8,9,10,11].

Cancer cells may develop altered cell-death pathways and mitochondrial functions, allowing them to escape apoptosis [12]. Mitochondria are bioenergetic, biosynthetic and signaling organelles, serving as important cellular stress sensors and contributing to cellular adaptation to the environment [13]. The major function of mitochondria is ATP production. However, they also regulate cell death and several cell signaling processes following the generation of reactive oxygen species (ROS), redox molecules and low molecular weight metabolites. Several lines of evidence show that mitochondria drive metabolic cancer cell reprogramming. In this regard, the adaptative metabolic changes in MM cells are emerging as the basis for PI resistance [8,14,15]. In particular, rewired glucose metabolism and increased antioxidant activity sustains the resistance to BTZ of resistant MM cells [14], and an increased expression of proteins involved in redox and energy metabolism has been found in BTZ-resistant cells [16]. Cancer cells often display a metabolic adaptation in order to maintain high ATP levels, increasing glycolytic rates and lactate production, despite the availability of oxygen (i.e., the Warburg effect) and efficiently functional mitochondria. Consistently, Maiso and colleagues demonstrated that increased glycolysis leads to tumor growth and bortezomib resistance in MM cells [8]. Furthermore, not only do many tumor cells that use this metabolism have functional mitochondria—with some cancer subtypes actually depending on mitochondrial respiration [13]—but the PI resistant phenotype shows an increase in both mitochondrial biomass and reliance on mitochondrial respiration [15,17]. Multiple aspects of mitochondrial bioenergetics—including mitochondrial biogenesis and turnover, cell death susceptibility and oxidative stress—support the flexibility of cancer cells to respond to changes in the microenvironment, allowing their survival under adverse conditions such as during chemotherapeutic and targeted cancer treatments.

In this study, we aimed at characterizing the metabolic phenotype associated with BTZ resistance in two different MM cell lines, BTZ-sensitive U266-S and BTZ-resistant U266-R, under both untreated and BTZ-treated conditions. The results demonstrated that BTZ resistance in MM cells is not only related to mitochondrial-dependent energy metabolism and powerful antioxidant defenses but is associated with a remarkably high activity of the protein glycosylation pathway, which may be, therefore, a potential target for overcoming BTZ resistance.

## 2. Materials and Methods

### 2.1. Cell Culture

U266-S and U266-R cells were grown in Roswell Park Memorial Institute (RPMI) 1640 medium supplemented with 20% foetal bovine serum and 1% penicillin-streptomycin. U266-R cells were selected for BTZ resistance by exposure to progressively higher concentrations of BTZ and attained resistance to higher doses of the drug after a quiescent state for a period of about 1 month. U266-S and U266-R were analyzed to evaluate apoptosis, proteasome activity, concentrations of low molecular weight compounds, ROS formation, mitochondrial membrane potential and mass, gene expression and ICAM1 expression, and to carry out transmission electron microscopy (TEM) determinations, after incubation for 24 h at 37 °C without or with 15 nM BTZ. 

### 2.2. 20S Proteasome Activity Assay

To evaluate proteasome activity, after washing, the cells were resuspended in culture medium at 3 × 10^5^ cells/well/100 µL in a 96 well plate. Proteasome activity assays were performed according to the assay kit’ instructions (Sigma-Aldrich, Mylan, Italy). Briefly, 25 µL of 400× Proteasome LLVY-R110 substrate was added to 10 mL of Assay Buffer (Proteasome Assay Loading Solution). The cells were then incubated with 100 µL/well of Proteasome Assay Loading Solution. Fluorescence intensities were measured by using the VICTOR3 Multilabel Plate Reader (Perkin Elmer, Monza, Mi, Italy) using 490-nm excitation and 525-nm emission filters.

### 2.3. Sample Preparation and Chromatographic Conditions of the HPLC Analysis of Metabolites

All ultrapure standards used for the evaluation of cellular metabolic profiles, tetrabutylammonium hydroxide and potassium di-hydrogen phosphate (KH_2_PO_4_) suitable for all buffer preparations were purchased from Sigma-Aldrich (St. Louis, MO, USA) and diluted in Ultrapure water (18.3 MΩ cm) (Milli-Q Synthesis A10, Millipore, Burlington, MA, USA). HPLC-grade methanol, far-UV acetonitrile and chloroform were supplied by J.T. Baker Inc. (Phillipsburgh, NJ, USA).

Metabolic analysis was performed after the deproteinization of cell samples (3 × 10^6^ cells) according to a protocol suitable for obtaining protein-free extracts for the further HPLC analysis of acid labile and easily oxidizable compounds [18]. The cells were washed twice with PBS at pH 7.4 and collected by centrifugation at 1860× *g* for 5 min at 4 °C. The cell pellets were deproteinized with the addition of 1 mL of ice-cold, nitrogen-saturated, 10 mM KH_2_PO_4_ in CH_3_CN, pH 7.4 (1:3, *v*/*v*). After vigorous mixing for 60 s, the samples were centrifuged at 20,690× *g* for 10 min at 4 °C. The organic solvent was removed from the deproteinized supernatants by two washes with 5 mL of chloroform. The upper aqueous phase, obtained by centrifugation under the same conditions, was then used for the HPLC analysis of low molecular weight metabolites. The simultaneous separation of 50 low molecular weight metabolites related to energy metabolism, oxidative/nitrosative stress and antioxidants—and including high energy phosphates (ATP, ADP, AMP, GTP, GDP, GMP, UTP, UDP, UMP, CTP, CDP, CMP and IMP), oxidized and reduced nicotinic coenzymes (NAD^+^, NADH, NADP^+^ and NADPH), glycosylated UDP-derivatives (UDP-galactose, UDP-glucose, UDP-*N*-acetyl-glucosamine and UDP-*N*-acetyl-galactosamine), reduced glutathione (GSH), nitrite and nitrate, and purines and pyrimidines (hypoxanthine, xanthine, uric acid, guanosine, uracil, β-pseudouridine and uridine)—were carried out using a Hypersil C-18, 250 × 4.6 mm, 5 µm particle size column, provided with its own guard column (Thermo Fisher Scientific, Rodano, Milan, Italy), following slight modifications of previously established ion pairing HPLC methods [19,20]. The HPLC apparatus was a SpectraSYSTEM P4000 pump (Thermo Fisher Scientific) interfaced to a highly-sensitive UV6000LP diode array detector (Thermo Fisher Scientific), equipped with a 5 cm light path flow cell and set up between 200 and 300 nm wavelength. Assignments and calculations of the aforementioned compounds in cell extracts were performed by comparing the retention times, absorption spectra, and areas of the peaks (calculated at the 260 nm wavelength for all compounds but GSH, nitrite and nitrate, which were calculated at the 206 nm wavelength) of the chromatographic runs of mixtures containing known concentrations of ultrapure standards.

### 2.4. ROS Evaluation

The levels of ROS were detected using the 2′,7′-dichlorofluorescein (DCF) assay (Sigma-Aldrich). Briefly, cells were labelled with 5 µM DCF and incubated for 10 min at 37 °C. After washing, the fluorescence intensity was measured according to the fluorescence detection conditions of FITC by using flow cytometry.

### 2.5. Measurement of Mitochondrial Membrane Potential 

A membrane potential probe, the DiOC2(3) (3,3’-Diethyloxacarbocyanine Iodide), was used to evaluate the mitochondrial membrane potential. Cells were incubated with 10 μM DiOC2(3) (Thermo Fisher Scientific, Milan, Italy) for 30 min at 37 °C, washed twice and resuspended in PBS for flow cytometry analysis. The intensity of green fluorescence of DiOC2(3) was detected using the MACSQuant Analyzer.

### 2.6. Mitochondrial Mass Evaluation

To determine mitochondrial mass, cells were reacted with 200 nM MitoTracker Red CMXRos probe (Thermo Fisher Scientific, Mylan, Italy) for 30 min at 37 °C, according to the manufacturer’s instructions. After being washed twice, the cells were analyzed by flow cytometry to quantify labelled mitochondria.

### 2.7. RT-qPCR 

For gene expression analysis, reverse transcription was performed on RNA extracted from cell samples by using High Capacity cDNA Reverse Transcription Kit (Thermo Fisher Scientific). The relative transcription of the genes of interest was determined by RT-qPCR using the Brilliant III Ultra-Fast SYBR Green QPCR Master Mix (Agilent Technologies) and 7900HT Fast Real-Time PCR System (Thermo Fisher). The expression of the following human genes was evaluated: PGC1α (Fw: ATGAAGGGTACTTTTCTGCCCC; Rw: GGTCTTCACCAACCAGAGCA) (207 bp, NM_001330751.2); SIRT1 (Fw: TGCTGGCCTAATAGAGTGGCA; Rw: CTCAGCGCCATGGAAAATGT) (102 bp, NM_012238.5); NAMPT (Fw: TGGCCTTGGGATTAACGTCT; Rw: CAAAATTCCCTGCTGGCGTC) (101 bp, NM_005746.3); SIRT3 (Fw: TCCGGAGGACTCCTTGGACTG; Rw: TCCCCGGCGATCTGAAGT) (367 bp, NM_001017524.2); OGA (Rw: TGCAACTTGCCTACTCATCAC, Fw: TTTCTGGGCCCGTACAAAGG) (177 bp, NM_001142434.1), OGT (Fw: ACAGCACAGAACCAACGAAAC -3’; Rw: GCTCAATTGCCTCCT GCAAC) (303 bp, NM_181672.3); ATP5B (Fw: AGCTCAGCTCTTACTGCGG; Rw: GGTGGTAGTCCCTCATCAAACT) (160 bp, NM_001686.4); CYTB (Fw TCCTCCCGTGAGGCCAAATATCAT; Rw AAAGAATCGTGTGAGGGTGGGACT) (160 bp NC_012920.1 [21]); OPA1 (Fw: CTGTGGCCTGGATAGCAGAA; Rw: GCGAGGCTGGTAGCCATATT) (291 bp, NM_001354663.2); MFN2 (Fw: GCTCGGAGGCACATGAAAGT; Rw: ATCACGGTGCTCTTCCCATT) (63 bp, NM_014874.4); DNM1L (Fw: TGGGCGCCGACATCA; Rw: GCTCTGCGTTCCCACTACGA), (54 bp, NM_012062.5); FIS1 (Fw TACGTCCGCGGGTTGCT; Rw CCAGTTCCTTGGCCTGGTT) (60 bp, NM_016068.3); GSTK1 (Fw: CTGGGCTTCGAGATCCTGTG; Rw: GGCAGACAAACTTCCACTGTC) (103 bp, NM_015917.3); B2M (Fw: AGCAGCATCATGGAGGTTTG; Rw: AGCCCTCCTAGAGCTACCTG) (229 bp, NM_004048.3); GAPDH (Fw: AATGGGCAGCCGTTAGGAAA; Rw: GCCCAATACGACCAAATCAGAG) (166 bp, NM_001256799.2). For each sample, the relative expression level of each studied mRNA was determined by comparison with the control housekeeping genes B2M and GAPDH using the 2^−ΔΔCt^ method.

### 2.8. ICAM1 Expression

ICAM1 expression was evaluated by flow cytometry using an anti-human ICAM1-PE antibody (clone HA58; Biolegend, San Diego, CA) on a MACSQuant Analyzer (Miltenyi Biotech) according to the manufacturer’s instructions.

### 2.9. Transmission Electron Microscopy

For conventional electron microscopy (EM), cells were pelleted and processed as described. Briefly, ultrathin sections were contrasted with uranyl acetate and lead citrate and observed with a JEOL JEM 2010 transmission electron microscope (Houghton, MI, USA) with a LaB6 thermoionic source operating at an acceleration voltage of 200 kV. In conventional EM analyses, micrographs of randomly selected cells were digitalized.

### 2.10. Statistical Analysis

Principal component analysis (PCA) was performed as previously described [22,23,24]. Explained variances are reported as a biplot of variables and key colored arrows representing the contribution of variables as square cosine (Cos^2^). The analyses were performed using the RStudio software using metabolomic data. The statistical analysis of the data was performed using the two-tailed Student’s t-test for independent samples for comparison of *n* = 2 groups and one-way ANOVA and the Holm–Sidak multiple comparisons test for *n* > 2 groups. Differences with values of *p* < 0.05 were considered statistically significant.

## 3. Results

### 3.1. Mitochondrial Biogenesis, Mitochondrial Dynamics and the Antioxidant System are Increased in U266-R

**Figure 1:**
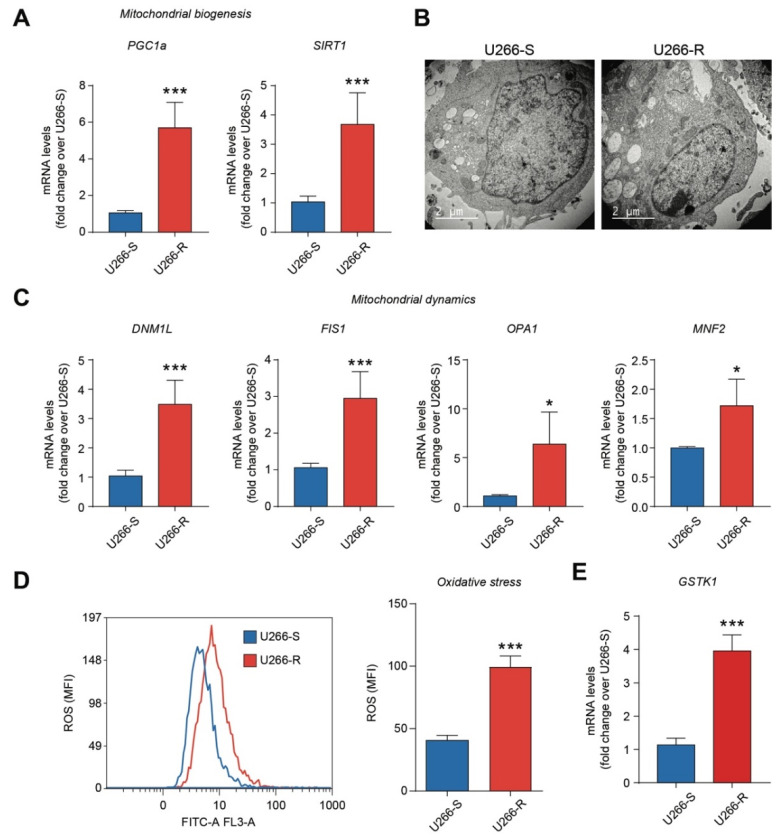
Mitochondrial biogenesis, mitochondrial dynamics and the antioxidant system are increased in U266-R. (**A**) Mitochondrial biogenesis analysis of mRNA levels of PGC1α and Sirtuin 1 (SIRT1) in U266-S versus U266-R cell lines; data are fold changes over U266-S and expressed as mean ± SEM of *n* ≥ 3 biological replicates; *** *p*-value < 0.001 versus U266-S. (**B**) Representative transmission electron microscopy (TEM) pictures of ultrathin sections showing mitochondrial contents of U266-S and U266-R cells. (**C**) Mitochondrial dynamics analysis of mRNA levels of DNM1L, FIS1, OPA1 and MNF2 in U266-S versus U266-R cell lines; data are fold changes over U266-S and expressed as mean ± SEM of *n* ≥ 3 biological replicates; * *p*-value < 0.05 and *** *p*-value < 0.001 versus U266-S. (**D**) Reactive oxygen species production during drug treatment was measured by the oxidation of 2′,7′-dichlorofluorescein (DCF) using flow cytometry. Representative plot and quantification of cytofluorimetric analysis in U266-S and U266-R cell lines; data are expressed as mean MFI ± SEM of *n* ≥ 3 biological replicates; *** *p*-value < 0.001 versus U266-S. (**E**) mRNA levels of GSTK1 in U266-S versus U266-R cell lines; data are fold change over U266-S and expressed as mean ± SEM of *n* ≥ 3 biological replicates; *** *p*-value < 0.001 versus U266-S. MFI: mean fluorescence intensity.

We first evaluate the activity of the ubiquitin–proteasome system in U266-R versus U266-S cells, finding that under basal conditions, it was significantly increased in U266-R compared to in U266-S (*p* < 0.001, Figure S1). As proteasome inhibition activates the UPR and ER stress, regulating mitochondrial morphology [25], we tested whether BTZ resistance in U266-R was mediated by increased values of different mitochondrial morpho-functional parameters. The results illustrated in Figure 1A demonstrate that the mitochondrial biogenesis markers PGC1α (peroxisome proliferator-activated receptor-γ coactivator α) and SIRT1 (Sirtuin 1) in U266-R were 6- and 4-fold higher, respectively, than the corresponding values determined in U266-S (*p* < 0.001). TEM images confirmed that this phenomenon was very likely responsible for the increased number of mitochondria in U266-R cells as compared to in U266-S cells (Figure 1B). 

Since mitochondria are the major intracellular source of ROS and that mitochondrial dynamics are tightly coupled to ROS generation, we performed ROS quantification in the two cell lines using cytofluorimetric analysis (Figure 1D). The results revealed higher levels of ROS in U266-R as compared to those detected in U266-S (*p* < 0.001, Figure 1D). To counteract the increase in intracellular ROS formation caused by elevated mitochondrial functions, Figure 1E shows that U266-R over-expressed the antioxidant enzyme GSTK1 (glutathione S-transferase pi 1) compared to the expression measured in U266-S (*p* < 0.001, Figure 1D). Furthermore, the quantification of GSH (Table S1) indicates that BTZ-resistant cells had about 1.5 times higher concentrations than those measured in BTZ-sensitive cells (*p* < 0.05). Hence, the increase in the main intracellular hydrophilic antioxidant provides U266-R with a better protection of free protein –SH groups, as well as supporting the adequate activity of various GSH-dependent enzymes involved in antioxidant defenses (GSH peroxidase and GSH reductase) and detoxification processes (GSH S-transferases). In addition to the better antioxidant status described above, U266-R showed lower rates of NO generation, as clearly indicated by the 5.9- and 2.0-fold decreases in nitrite and nitrite+nitrate concentrations, respectively, in comparison to the concentrations detected in U266-S cells (*p* < 0.05; Table S1).

### 3.2. U266-R Cells Exhibit Increased Concentrations of GTP, UTP and CTP 

As shown in Table S2, differences in adenine nucleotide concentrations, ECP and the ATP/ADP ratio were found between U266-S and U266-R deproteinized cell extracts, thus indicating the equal mitochondrial phosphorylating capacity (ATP/ADP) of the two clones. Quantification of the other purine (GTP, GDP, GMP and IMP) and pyrimidine (UTP, UDP, UMP, CTP, CDP and CMP) nucleotides (Table S3) evidenced that the BTZ-resistant clone had significantly higher GTP, UTP and CTP concentrations compared to the U266 BTZ-sensitive clone. However, for UMP, no differences were observed when comparing diphosphorylated and monophosphorylated purine and pyrimidine nucleosides. The significantly lower UMP values found in U266-R might be related not only to higher UTP values but also to the overall increase in UDP derivatives characterizing the resistant clone.

### 3.3. Redox State of Nicotinic Coenzymes in Bortezomib Sensitive and Resistant Cells

In strict connection with the energy state, we evaluated the concentrations of NAD^+^, NADH, NADP^+^ and NADPH in the two clones. Among the four forms of nicotinic coenzymes, significant differences were detected only in the case of NADPH, the concentration of which was lower in U266-S (0.068 ± 0.004 nmol/10^6^ cells) than in U266-R (0.085 ± 0.007 nmol/10^6^ cells; *p* < 0.05, Table S4). The sum of NAD^+^ and NADH in U266-S and U266-R was 1.45 ± 0.15 and 1.69 ± 0.29 nmol/10^6^ cells, respectively, whilst that of NADP^+^ and NADPH was 0.232 ± 0.022 and 0.209 ± 0.010 nmol/10^6^ cells, respectively. In consequence of the similar NAD^+^ and NADH concentrations, their ratio was not different between the two clones. Conversely, given the higher NADPH and the equal NADP^+^ + NADPH values recorded in U266-R, the NADP^+^/NADPH ratio was significantly higher in U266-S (2.07 ± 0.11) than in U266-R (1.75 ± 0.21), suggesting higher biosynthetic capacities/needs in the BTZ-resistant clone. 

### 3.4. Bortezomib Resistance is Associated with Increased Glycosylation 

**Figure 2:**
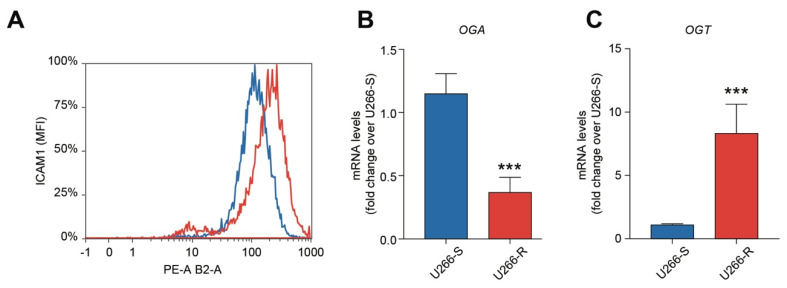
U266-R showed increased glycosylation. (**A**) Representative plot of ICAM1 cytofluorimetric analysis in U266-S and U266-R cell lines. (**B**,**C**) mRNA levels of O-GlcNAc transferase (OGA) and O-GlcNAc transferase (OGT) in U266-S versus U266-R cell lines; data are fold changes over U266-S and expressed as mean ± SEM of *n* ≥ 3 biological replicates; *** *p*-value < 0.001 versus U266-S.

Using our analytical HPLC method, it was possible to separate and quantify the four main forms of glycosylated UDP-containing compound, namely UDP-galactose (UDP-Gal), UDP-glucose (UDP-Glc), UDP-*N*-acetyl-galactosamine (UDP-GalNac) and UDP-*N*-acetyl-glucosamine (UDP-GlcNac). Compared to the values measured in U266-S, BTZ-resistant cells showed increased levels of UDP-Gal, UDP-Glc, UDP-GalNac and UDP-GlcNac, of about 1.79, 1.39, 1.51 and 1.81 times, respectively (*p* < 0.05; Table S5). Consistent with these findings, U266-R showed increased cell surface expression of ICAM1/ICAM-1 (intercellular adhesion molecule 1, Figure 2A), an inducible glycoprotein whose expression is regulated by glycosylation levels [26]. These results demonstrated that higher activity of the hexosamine biosynthetic pathways (HBP) and pronounced and efficient protein glycosylation processes are associated with BTZ resistance. To confirm these observations, we analyzed the expression of the genes encoding for the O-GlcNAc transferase (OGT) and O-GlcNAcase (OGA), i.e., the enzymes responsible for the glycosylation and de-glycosylation of proteins through linkage and removal, respectively, of a GlcNAc moiety [27]. We found that OGT and OGA levels were higher and lower, respectively, in U266-R than in U266-S (Figure 2B).

### 3.5. Mitochondrial Fitness after BTZ Challenge Contributes to Drug Resistance 

**Figure 3:**
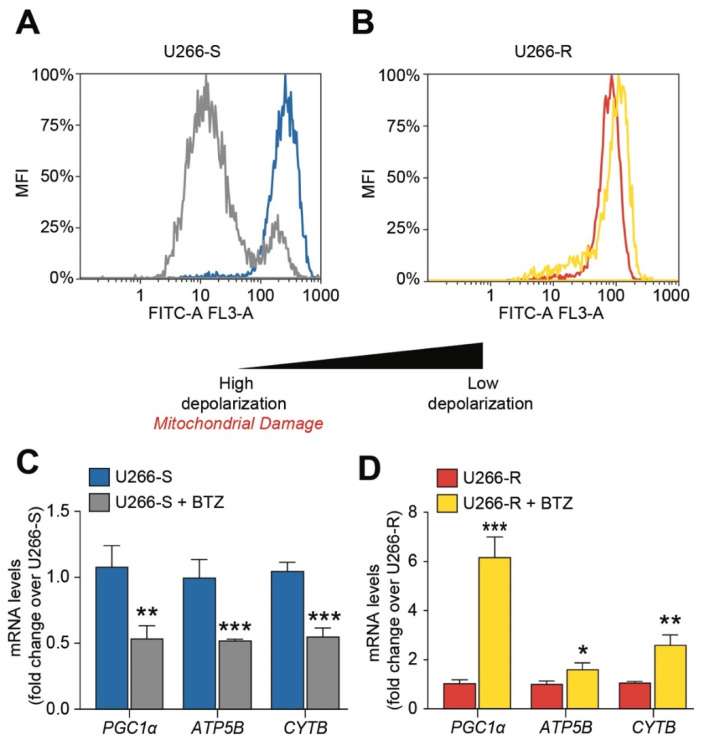
Mitochondrial fitness after bortezomib (BTZ) challenge contributes to drug resistance. (**A**,**B**) Representative plots of mitochondrial depolarization in untreated U266-S and U266-S + BTZ (**A**) and untreated U266-R and U266-R + BTZ (**B**). (**C**) mRNA levels of PGC1α, ATP synthase (ATP-synt) and CytB in untreated U266-S and U266-S + BTZ cell lines; data are fold changes over untreated U266-S and expressed as mean ± SEM of *n* ≥ 3 biological replicates; ** *p*-value < 0.01 and *** *p*-value < 0.001 versus U266-S. (**D**) mRNA levels of PGC1α, ATP-synt and CytB in untreated U266-R and U266-R + BTZ cell lines; data are fold changes over untreated U266-R and expressed as mean ± SEM of *n* ≥ 3 biological replicates; * *p*-value < 0.05, ** *p*-value < 0.01 and *** *p*-value < 0.001 versus U266-R.

Upon analyzing the effect of BTZ treatment on mitochondrial polarization status by flow cytometry, we noted that BTZ-treated U266-S showed high mitochondrial depolarization compared to untreated U266-S (Figure 3A). To the contrary, U266-R did not display differences before and after BTZ exposure (Figure 3B). Mitochondrial depolarization was accompanied in U266-S by a significative decrease in PGC1α (*p* < 0.01) and a down-regulation of cytochrome b (Cyt-B) and ATP synthase (ATP-synt), indicating decreased efficiency of both the electron transport chain (ETC) and oxidative phosphorylation (OXPHOS, Figure 3C). To the contrary, BTZ-treated U266-R up-regulated the above-described genes as a protective mechanism to defend mitochondria against PI treatment (Figure 3D).

### 3.6. Antioxidant Status and NO Metabolism in U266-S and U266-R after Exposure to BTZ 

**Figure 4:**
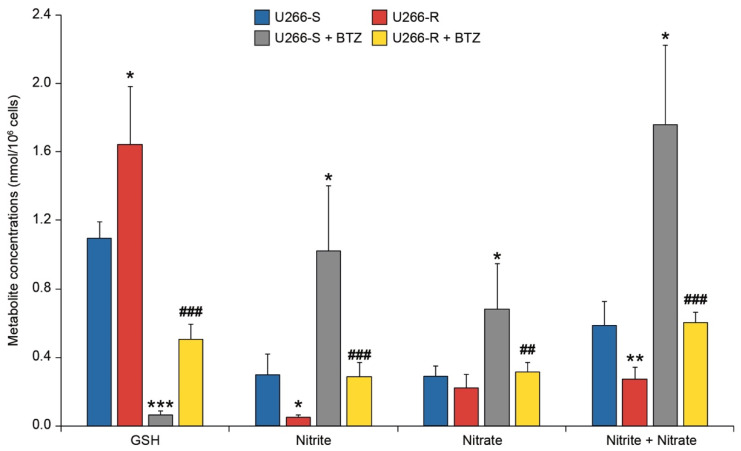
Concentrations of glutathione (GSH) (representative of the cell antioxidant status), nitrite and nitrate (as indicators of nitrosative stress) in sensitive versus resistant U266 exposed to BTZ. Metabolite concentrations are expressed as nmol per 10^6^ cells in U266-S and U266-S + BTZ and in U266-R and U266-R + BTZ. Data are shown as mean ± SEM of *n* = 4 biological replicates; * *p*-value < 0.05, ** *p*-value < 0.01 and *** *p*-value < 0.001 versus U266-S; ^##^ *p*-value < 0.01 and ^###^ *p*-value < 0.001 versus U266-S + BTZ.

The effect of BTZ on the concentrations of GSH, nitrite, nitrate and nitrite+nitrate are illustrated in Figure 4. Compared to the corresponding untreated cells, we observed a depletion of GSH levels of about 94% and 69% in U266-S and U266-R after exposure to BTZ (*p* < 0.05, Figure 4). It is, however, worth underlining that BTZ-treated U266-R had five times more GSH than BTZ-treated U266-S (*p* < 0.05). Consequently, following BTZ treatment, higher concentrations of nitrite (1.02 ± 0.39 nmol/10^6^ cells) and nitrate (0.684 ± 0.263 nmol/10^6^ cells) were found in U266-S, in comparison with the corresponding values (0.289 ± 0.083, 0.316 ± 0.056 and 0.605 ± 0.060 nmol/10^6^ cells) detected in U266-R (*p* < 0.05).

### 3.7. Cell Energy State in U266-S and U266-R after Exposure to BTZ

**Figure 5:**
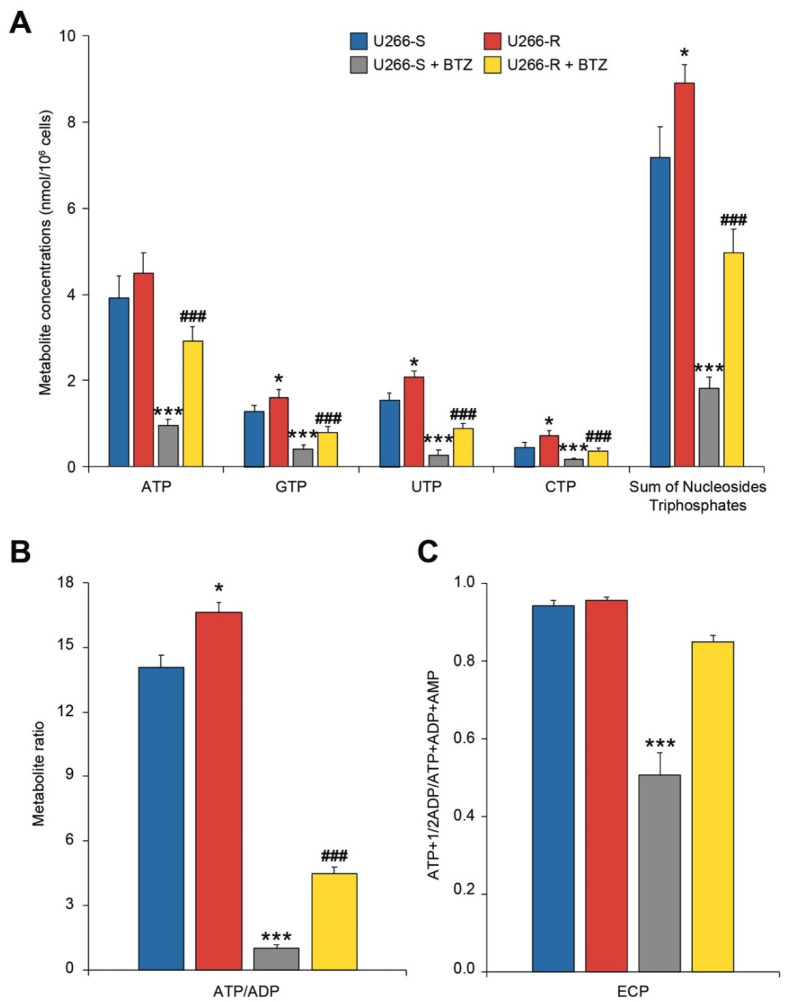
Cell energy states in U266-S and U266-R after exposure to BTZ. **A**–**C**) Metabolite concentrations expressed as nmol per 10^6^ cells (**A**), ATP/ADP ratio (**B**) and Energy Charge Potential (ECP) (**C**), in U266-S and U266-S + BTZ and in U266-R and U266-R + BTZ. Data are shown as mean ± SEM of *n* = 4 biological replicates; * *p*-value < 0.05 and *** *p*-value < 0.001 versus U266-S; ^###^ *p*-value < 0.001 versus U266-S + BTZ.

BTZ treatment also led to significant changes in energy metabolism, even though those occurring to U266-R were remarkably attenuated compared to those taking place in U266-S. Decreases of 75%, 62%, 83%, 61% and 75% in the concentrations of ATP, GTP, UTP, CTP and total nucleoside triphosphates, respectively, were recorded following BTZ exposure in U266-S (*p* < 0.05) in comparison to in untreated cells (Figure 5A). The aforementioned metabolites in U266-R decreased after BTZ treatment by 35%, 50%, 58%, 50% and 46%, respectively, and were significantly lower than the corresponding values measured in untreated U266-R (*p* < 0.05), although significantly higher than the values determined in BTZ-treated U266-S (*p* < 0.05, Figure 5A). Moreover, the concentrations of AMP and ADP in U266-S increased after BTZ treatment, respectively, from 0.103 ± 0.045 to 0.934 ± 0.186 and from 0.282 ± 0.052 to 0.997 ± 0.130 nmol/10^6^ cells (3.7 and 13.2 times higher than the values in untreated U266-S, respectively, *p* < 0.05, Table S6). The concentrations of the aforementioned adenine nucleotides in BTZ-treated U266-R were 0.668 ± 0.137 nmol/10^6^ cells in the case of ADP (2.4 times higher than the values in untreated U266-R, *p* < 0.05) and 0.238 ± 0.056 nmol/10^6^ cells (2.3 times higher than the values in untreated U266-R, *p* < 0.05). The levels of both ADP and AMP in the BTZ-treated resistant clone were significantly lower than those measured in the BTZ-treated sensitive clone (*p* < 0.05, Table S6). The imbalance in the concentrations of adenine nucleotides induced by BTZ treatment was due to mitochondrial dysfunction causing decreases in their phosphorylating capacity (decreases in the ATP/ADP ratio) and an overall energetic suffering (decrease in the energy charge potential—ECP values, Figure 5B). However, whilst the ATP/ADP ratio decreased by 11 times (from 14.1 ± 2.6 to 1 ± 0.3) in U266-S following BTZ treatment, U266-R showed only a reduction of about 3.7 times (from 16.6 ± 0.7 before to 4.5 ± 0.6 after BTZ exposure). Similarly, the ECP values following BTZ challenge decreased by 1.86 times in U266-S (from 0.942 ± 0.013 before to 0.508 ± 0.057 after BTZ treatment) and only 1.12 times in U266-R (from 0.955 ± 0.001 to 0.850 ± 0.015 after treatment, Figure 5C). 

### 3.8. Redox State of Nicotinic Coenzymes in U266-S and U266-R after Exposure to BTZ

**Figure 6:**
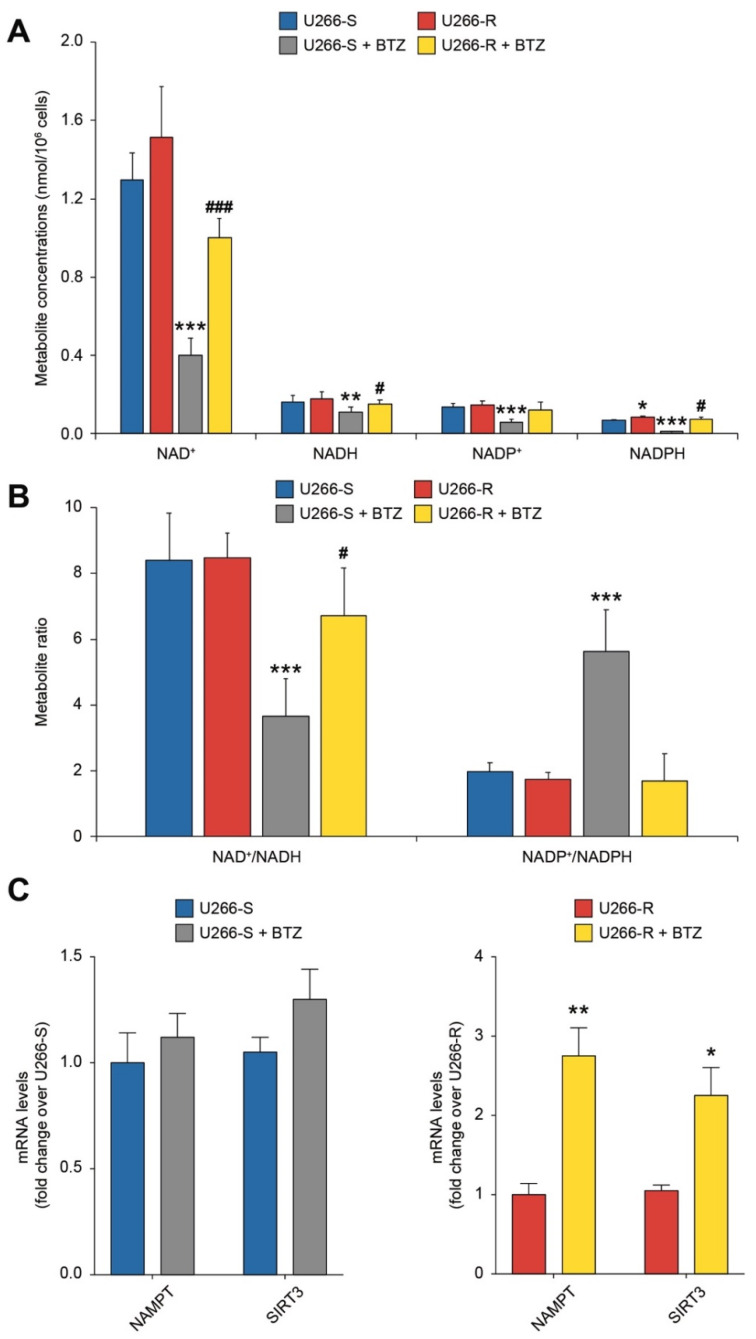
Redox state of nicotinic coenzymes in U266-S and U266-R after exposure to BTZ. (**A**,**B**) Metabolite concentrations expressed as nmol per 10^6^ cells (**A**) and NAD^+^/NADH and NADP^+^/NADPH ratios (**B**) in U266-S and U266-S + BTZ and in U266-R and U266-R + BTZ. Data are shown as mean ± SEM of *n* = 4 biological replicates; * *p*-value < 0.05 and *** *p*-value < 0.001 versus U266-S; ^#^ *p*-value < 0.05 and ^###^ *p*-value < 0.001 versus U266-S + BTZ. (**C**) mRNA levels of NAMPT and SIRT3 in untreated U266-S, U266-S + BTZ, U266-R and U266-R + BTZ cell lines; data are fold changes over untreated U266-S or U266-R and expressed as mean ± SEM of *n* ≥ 3 biological replicates; * *p*-value < 0.05 and ** *p*-value < 0.01 versus U266-R.

Using our HPLC methods, allowing the simultaneous separation and quantification of both oxidized (NAD^+^ and NADP^+^) and reduced (NADH and NADPH) nicotinic coenzymes, it was possible to observe strikingly different effects on their respective concentrations after BTZ treatment (Figure 6A). 

Compared to in untreated cells, the concentrations of NAD^+^, NADH, NADP^+^ and NADPH decreased, respectively, by 69%, 29%, 55% and 84% in BTZ-treated U266-S, and by 34%, 15%, 19% and 13% in U266-R, after BTZ treatment (Figure 6A). As a consequence of such changes in the absolute concentrations, it was found that whilst BTZ remarkably modified the oxidized/reduced ratios of nicotinic coenzymes in U266-S (60% decrease in NAD^+^/NADH and 286% increase in NADP^+^/NADPH), these effects in U266-R were definitely modest in the case of NAD^+^/NADH (20% decrease) and even negative in the case of NADP^+^/NADPH (13% decrease, Figure 6B). 

In accordance with the modest decrease in the NAD^+^/NADH ratio in BTZ-treated U266-R, we observed a significant up-regulation of NAMPT and SIRT3 in resistant cells after drug exposure (Figure 6C). No change was observed in BTZ-treated U266-S compared to in untreated cells (Figure 6C). 

### 3.9. Glycosylated UDP-Derivatives in U266-S and U266-R after Exposure to BTZ

**Figure 7:**
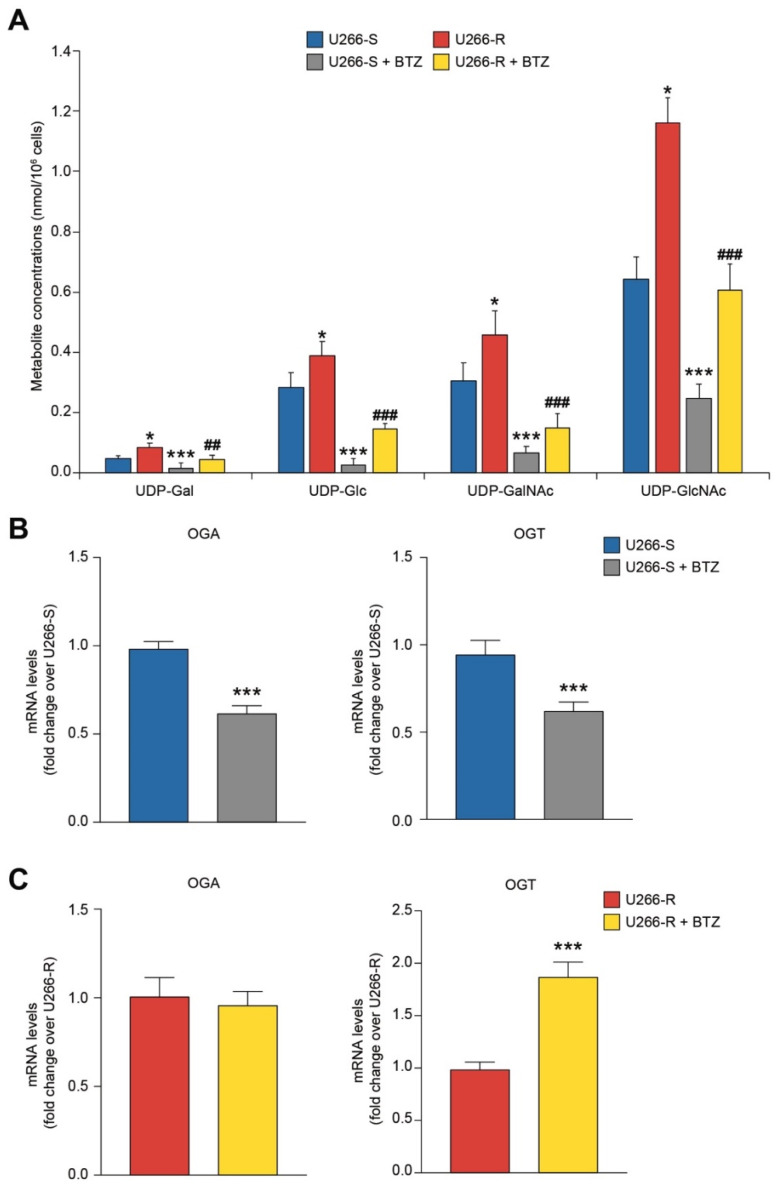
Glycosylated UDP derivatives in U266-S and U266-R after exposure to BTZ. (**A**) Metabolite concentrations are expressed as nmol per 10^6^ cells in U266-S and U266-S + BTZ and in U266-R and U266-R + BTZ. Data are shown as mean ± SEM of *n* = 4 biological replicates; * *p*-value < 0.05 and *** *p*-value < 0.001 versus U266-S; ^##^ *p*-value < 0.01 and ^###^ *p*-value < 0.001 versus U266-S + BTZ. (**B**,**C**) mRNA levels of OGA and OGT in untreated U266-S and U266-S + BTZ (**B**) and in U266-R and U266-R + BTZ (**C**); data are fold changes over untreated U266-S (**B**) or over untreated U266-R (**C**) and expressed as mean ± SEM of *n* ≥ 3 biological replicates; *** *p*-value < 0.001 versus U266-S or U266-R.

A particular vulnerability of U266-S to BTZ toxicity was observed for UDP-Gal (−65%), UDP-Glu (−90%), UDP-GalNac (−78%) and UDP-GlcNac (−62%), mainly involved as metabolites (UDP-Gal and UDP-Glu) and end products (UDP-GalNac and UDP-GlcNac) of the hexosamine biosynthetic pathway (HBP, Figure 7A). Their concentrations were significantly lower than those measured in untreated U266-S (*p* < 0.05). The U266-R clone exhibited less evident decreases in the aforementioned compounds after BTZ exposure (UDP-Gal, −48%; UDP-Glu, −62%; UDP-GalNac, −67%; UDP-GlcNac, −48%; *p* < 0.05 compared to both untreated U266-R and BTZ-treated U266-S), indicating that protein glycosylation processes are operative even under exposure to BTZ. Notably, the concentration of UDP-GlcNac in U266-R after 24 h incubation with BTZ (0.606 ± 0.086 nmol/10^6^ cells) was not different from that measured in untreated U266-S (0.641 ± 0.074 nmol/10^6^ cells). In accordance with these results, we found that U266-R up-regulated OGT at 24 h after BTZ treatment. To the contrary, the sensitive clone down regulated both OGA and OGT (Figure 7B,C).

### 3.10. Purine and Pyrimidine Compounds in U266-S and U266-R before and after Exposure to BTZ

The data summarized in Table S7 refer to compounds derived from the metabolism of purines (IMP, Hyp, guanosine) and pyrimidines (UMP, uridine, uracil, β-pseudouridine) recorded in the BTZ-sensitive and -resistant clones before and after PI treatment. Upon comparing untreated U266-S and U266-R, only UMP and uracil had different concentrations between the cell extracts of the two clones (*p* < 0.05). Following the incubation with BTZ, striking differences in the concentrations of all the aforementioned compounds between U266-S and U266-R were observed. The sensitive clone showed higher levels of these metabolites compared to the values measured in BTZ-treated U266-R (*p* < 0.05), suggesting increased degradation rates for adenine nucleotides (increases in IMP and Hyp), for UTP and glycosylated UDP-derivatives (UMP, uridine and uracil) and for RNA (β-pseudouridine).

### 3.11. Principal Component Analysis of BTZ’s Effects on Metabolite Levels in U266-S Versus U266-R Cells

**Figure 8:**
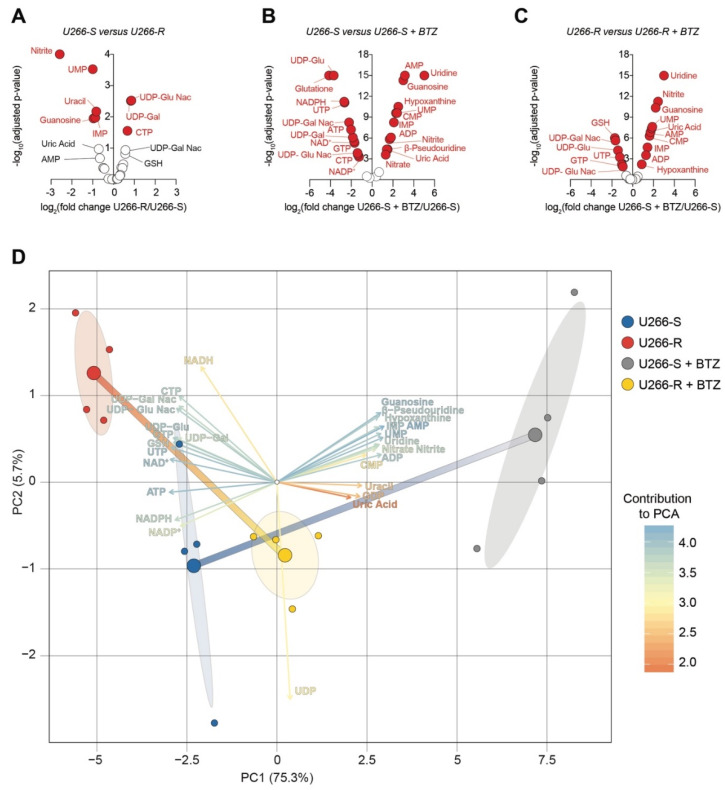
Principal component analysis on BTZ’s effects on metabolites levels in U266-S versus U266-R cells. (**A**–**C**) Volcano plots of metabolites showing −log10 of the adjusted *p*-value plotted against the log2 of the fold-change in metabolite levels in U266-R (**A**), U266-S + BTZ (**B**) and U266-R + BTZ (**C**) for *n* = 4 biological replicates. (**D**) Principal component analysis (PCA) biplot of metabolite levels for U266-S, U266-R, U266-S + BTZ and U266-R + BTZ (*n* = 4); key colored arrows represent the variables’ contribution to the PCA; dots indicate individual replicates and bigger dots, the mean points of groups. Confidence ellipses for clusterized groups are also shown.

To compare how these metabolites changed between sensitive and resistant U266 and the relative BTZ effects, we generated volcano plots for U266-S versus U266-R, U266-S versus U266-S + BTZ and U266-R versus U266-R + BTZ (Figure 8A–C). We found that among the most significantly differentially expressed metabolites in U266-R as compared to in U266-S, UDP-GluNac and UDP-Gal were the most representative (Figure 8A). Our analysis showed that after BTZ exposure, in U266-S cells—besides AMP, uridine, hypoxanthine, CMP, nitrite and nitrate accumulation—a great influence was held by UDP-Glu, UDP-GluNac, UDP-Gal and UDP-GalNac reduction (Figure 8B). Such a finding was coupled with a reduced BTZ impact on these metabolites in the U266-R clone (Figure 8C). We finally performed a principal component analysis (PCA), integrating individual metabolite levels, in order to highlight the multivariate effect of metabolites in sensitive versus resistant U266 clones and BTZ-induced effects. We generated two principal components (i.e., PC1 and PC2) explaining a total of 81% of the variances among groups (i.e., U266-S, U266-R, U266-S + BTZ and U266-R + BTZ, Figure 8D). 

The results showed that hypoxanthine, AMP, ATP, nitrite and nitrate were strictly correlated in discriminating U266-S versus U266-S + BTZ groups. Notably, the main contributors in U266-R versus U266-S clustering were UDP-Glu, UDP-Glu Nac, UDP-Gal and UDP-Gal Nac (Figure 8D). Such evidence was also confirmed in U266-R + BTZ as compared to U266-S + BTZ, thus sustaining the hypothesis that BTZ has less efficiency in reducing glycosylation processes in U266-R versus in U266-S cells (Figure 8D).

## 4. Discussion

Resistance to chemotherapy is one of the major issues that clinicians have to face when treating oncological patients with anticancer agents [28,29]. The administration of nearly any chemotherapeutic drug may meet drug resistance in a variable percentage of cancer patients, after a variable number of treatment cycles. The proteasome inhibitor BTZ rapidly became one of the most widely used drugs for the treatment of MM [30]. Unfortunately, many patients eventually relapse following BTZ therapy [31]. In general, compared to the parental sensitive cancer cell lines, the development of resistance towards chemotherapeutic toxicity involves changes in a vast number of gene expressions, protein expressions, and metabolic pathways and cycles [32]. 

In the present study, we were able to generate a human MM cell line resistant to BTZ, after an in vitro administration protocol, inducing a dramatic decrease in BTZ’s effectiveness towards U266 cells. The selection of this resistant U266-R clone allowed us to characterize the metabolic and molecular differences between the BTZ-sensitive and -resistant clones, either under basal conditions or after treatment with BTZ. First, we found that the difference in cell death was not caused by mutation or differences in the activity of the 20S proteasome between the two cell lines. Indeed, U266-R had increased proteasome activity compared to U266-S, but still BTZ was able to inhibit it, even if longer times of exposure to BTZ were required to obtain effects comparable to those found when using the sensitive clone. Proteasome inhibition is known to induce ER stress that can lead to apoptosis activation, with the transition of the UPR from a protective to an apoptotic response. The close contact between ER and mitochondria mutually impacts mitochondrial division and fusion dynamics [33], synthesis and transfer of lipids and the exchange of Ca^2+^. This last phenomenon regulates ER chaperones, mitochondrial ATP production and apoptosis [34]. Here, we demonstrated that U266-R had an increased number of mitochondria and up-regulated biogenesis markers such as PGC1α and SIRT1 [35,36,37,38,39]. The synergistic interaction with SIRT1 is reported to cause an increase in the PGC1α activity, subsequently leading to the promotion of mitochondrial OXPHOS [40]. 

The maintenance of a healthy mitochondrial network, fundamental to support cell energy demand, is strictly dependent on mitochondrial fission and fusion. These processes are considered as mitochondrial quality control, regulating changes in mitochondrial size and shape, mitochondrial growth and redistribution, as well as intervening in mitochondrial cristae remodeling in response to changes in cell energy requests [41]. Compared to the sensitive clone, U266-R showed increased mitochondrial dynamics, with the up-regulation of both fission (DNM1L and FIS1) and fusion (OPA1 and MFN2) genes, which encoded for GTPase proteins that shape the overall steady-state morphological and functional characteristics of mitochondria. In accordance with these results, we found higher levels of GTP in U266-R compared to in U266-S, indicating higher substrate availability in BTZ-resistant cells to support the increased activity of the mitochondrial quality control network. 

The increase in the mitochondrial number, dynamics and metabolism, as well as the increase in PGC1α—an up-regulator of the expression of most mitochondrial proteins—lead to the over-production of intracellular ROS [40]. However, in order to afford better protection against potential toxicity caused by higher ROS production, PGC1α can also induce the expression of genes regulating the synthesis of ROS-scavenging enzymes. In accordance with these observations, we found that higher ROS levels in U266-R were associated with an increased expression of antioxidant enzymes, such as GSTK1. Furthermore, U266-R had higher levels of GSH, allowing either the support of adequate GSTK1 activity, the counteraction of ROS-mediated cell injury or the prevention of mitochondrial biogenesis inhibition [42]. 

From the metabolic point of view, the most striking difference between U266-S and U266-R cells concerned the concentrations of glycosylated UDP-derivatives. On average, UDP-Gal, UDP-Glc, UDP-GalNac and UDP-GlcNac had 1.5-fold higher concentrations in U266-R than in U266-S. The production of UDP-Gal and UDP-Glc, requiring the consumption of significant amounts of UTP, is necessary for the synthesis of two corresponding *N*-acetylated forms through the HBP [43]. Hence, the HBP modulates the synthesis of UDP-GalNac and UDP-GlcNac depending on the cell’s metabolic requirements [44]. Both compounds participate in the formation of glycans and glycosaminoglycans [40]. The availability of adequate concentrations of UDP-GalNac and UDP-GlcNac ensures the post-translational *O*-linked and *N*-linked glycosylation of proteins, thus regulating a vast number of cell functions including cell signaling [45], mitochondrial functions [46] and apoptosis [47]. In the last few decades, it has been found that cancer cells have a higher activity of the HBP than non-tumoral cells [48] and that a highly efficient HBP is indicative of increased tumor malignancy [49,50] and resistance to chemotherapeutics [51]. An increase in HBP-mediated protein *O*-glycosylation has also been found in the case of MM [52], although data in this type of cancer are not particularly abundant. Recently, Luanpitpong et al. (2017) demonstrated that resistance to BTZ in mantle cell lymphoma is associated with high protein glycosylation and that the inhibition of OGA (the enzyme removing glycans from glycosylated proteins) is able to restore sensitivity to BTZ. In the HBP, OGT is the enzyme responsible for linking the GlcNac moiety of UDP-GlcNac to the OH-groups of specific Ser and Thr residues of the proteins to glycosylate, via the formation of an O-glycosidic bond and the production of free UDP. Vice versa, OGA is the enzyme catalyzing the hydrolysis of the *O*-glycosidic bond and the removal of GlcNAc from O-glycosylated proteins [27,53]. In the present study, we found that, compared to U266-S, U266-R cells under basal conditions have an increased expression of OGT. This finding, coupled with the higher concentrations of UDP-derivatives involved in the HBP, reinforce the hypothesis that resistance to BTZ is also mediated by efficient post-translational protein glycosylation. After BTZ exposure, U266-S dramatically decreased the concentrations of UDP-Gal, UDP-Glc, UDP-GalNac and UDP-GlcNac. Conversely, BTZ-treated U266-R maintained quite relevant concentrations of the aforementioned compounds, particularly of UDP-GlcNac, assuming a pattern of substrates for protein glycosylation similar to that observed in U266-S cells under basal conditions. To support the evidence for a central role of HBP in BTZ resistance, we also found that even after BTZ treatment, U666-R had elevated UTP concentrations that were indispensable to sustain the adequate activity of the HBP. In accordance with these observations, U266-R showed increased OGT expression after BTZ exposure; to the contrary, the dramatic decrease in glycosylated UDP-derivative concentrations was associated with the down-regulation of both OGT and OGA in the BTZ-sensitive clone. It is also worth underlining that U266-R preserved the concentrations of GTP and CTP during BTZ treatment. Both nucleotides function as fundamental energy substrate donors in the complex sequences of alternative pathways of protein glycosylation, the former being used in the synthesis of GDP-mannose and the latter, as a substrate of dolichol kinase. Hence, GTP and CTP availability ensure the synthesis of dolicholphosphate, which is actively involved in protein glycosylation [54]. 

To support the high energy expenditure of the HBP and protein glycosylation, mitochondrial-dependent energy metabolism is the second most evident characteristic distinguishing U266-R from U266-S. Particularly after treatment with BTZ, U266-R showed significantly higher values of the ATP/ADP ratio and of ECP, the first parameter representing a good indicator of the mitochondrial phosphorylating capacity [55] and the second one being indicative of cell energy wellness and functional mitochondria [56,57]. Cytofluorimetric analysis confirmed a strong mitochondrial depolarization only in BTZ-treated U266-S. No change was detected in resistant cells after PI treatment. Mitochondrial fitness damage in the sensitive clone was also associated with the down-regulation of the ATP-synt and CytB genes, as well as with the decreased expression of PGC1α. To the contrary, U266-R responded to BTZ by increasing CytB and PGC1α. Moreover, BTZ exposure also induced the up-regulation of SIRT3, which favours the effects of PGC1α on mitochondrial-related gene expression [40]. 

Comparing U266-S and U266-R, we found that only the NADPH concentration was higher in resistant cells; the absolute concentrations of oxidized (NAD^+^ and NADP^+^) and reduced (NADH and NADPH) coenzymes, as well as the NAD^+^/NADH ratio, were similar in the two clones under basal conditions. This similarity between U266-S and U266-R was lost under BTZ treatment, with U266-S undergoing a dramatic depletion of NAD^+^, NADP^+^ and NADPH, with a concomitant halving and doubling, respectively, of the NAD^+^/NADH and NADP^+^/NADPH ratios. These findings strongly support results reported by Cagnetta et al. indicating that BTZ in sensitive MM cells deeply interferes with NAD^+^ metabolism [58]. Conversely, we found that BTZ-treated U266-R had minimal decreases in both reduced and oxidized nicotinic coenzymes and in their relative ratios. Since cellular NAD^+^ depletion is strictly connected either to poly(ADP-ribose) polymerase (PARP) activity [59,60] or to NAD^+^-glycohydrolase [61], it is conceivable to hypothesize that U266-R cells have a low activity of both NAD^+^-consuming enzymes. Of interest, BTZ-treated U266-R increased the expression of NAMPT, encoding for a crucial enzyme in NAD^+^ biosynthesis and also involved in sirtuin activation [62]. These results are consistent with a previous report showing that the NAMPT/SIRT3/IDH2 (isocitrate dehydrogenase 2) axis is a major determinant of PI responsiveness [63]. IDH2 is a mitochondrial enzyme critical for the maintenance of the mitochondrial redox balance due to the formation of NADPH during the oxidative decarboxylation of isocitrate to 2-oxoglutarate. [64]. The combination of PI with NAMPT inhibitors induced the down-regulation of IDH2 and the Krebs cycle, leading to MM cell death [63]. The maintenance of a high NAD^+^ level is not only fundamental for the functioning of the ETC and OXPHOS but also allows the performance of specific oxido-reductive reactions crucial for the proliferation of cancer cells, such as serine synthesis [65]. The values of the NAD^+^/NADH ratio in BTZ-treated U266-S and U266-R, taken together with the corresponding values of the ATP/ADP ratio, are indicative of profound mitochondrial dysfunction in the sensitive clone and near-normal mitochondrial metabolism in the resistant clone. Additionally, the concomitant remarkable NADPH depletion and the high increase in the NADP^+^/NADPH ratio in U266-S after BTZ treatment seem to suggest the activation of NADPH-oxidase, one of the major non-mitochondrial ROS sources deeply involved in mediating cell oxidative stress [66]. This consideration is supported by the values of GSH in U266-S that, after BTZ treatment, decreased to nearly zero. Vice versa, BTZ-treated U266-R, although showing consistent GSH decrease compared to untreated cells, had almost three times higher GSH levels compared to the levels found in BTZ-treated U266-S. Interestingly, BTZ-sensitive cells under basal conditions showed higher concentrations of nitrite and nitrate than those measured in U266-R. This difference was even more evident after treatment with BTZ. Since nitrite and nitrate levels are stable end products of nitric oxide (NO), reflecting its cellular production, this suggests that resistance to BTZ is also connected to NO metabolism. It is plausible that BTZ-resistant cells have low basal levels of constitutive nitric oxide synthases (NOS), as well as a moderate BTZ-mediated induction of iNOS.

Our metabolic analyses in BTZ-sensitive and -resistant clones of U266 clearly showed that the mechanisms of resistance to this chemotherapeutic agent concern a complex strategy triggered by MM cells. This involves the capacity to control a wide spectrum of metabolic pathways and cell functions implicated in BTZ cytotoxicity, with the aim of optimizing any possible defense mechanism and of maintaining an almost unaltered proliferating capacity. It is worth underlining the role of the HBP and mitochondrial dynamics, which may represent crucial targets for overcoming drug resistance.

## 5. Conclusions

In conclusion, the cloning of a stable BTZ-resistant MM cell line that may represent a selective model to study, in depth, the mechanisms of resistance to BTZ appears highly relevant. Last but not least, the complexity of the cell strategy to maximize proliferation and improve the defense mechanisms leading to drug resistance strongly suggests that it would be useful to design improved studies aimed at testing multi-drug administration, targeting multiple sites connected to these two resistant cancer cell features.

## Supplementary Materials

The following are available online at https://www.mdpi.com/2218-273X/10/5/696/s1, Figure S1; Tables S1-S7.
