# Supplementary figures and images for: Mitochondrial Functions, Energy Metabolism and Protein Glycosylation are Interconnected Processes Mediating Resistance to Bortezomib in Multiple Myeloma Cells
Source: Biomolecules. 2020 Apr 30;10(5):696. doi: 10.3390/biom10050696 (PMC7277183; doi:10.3390/biom10050696)

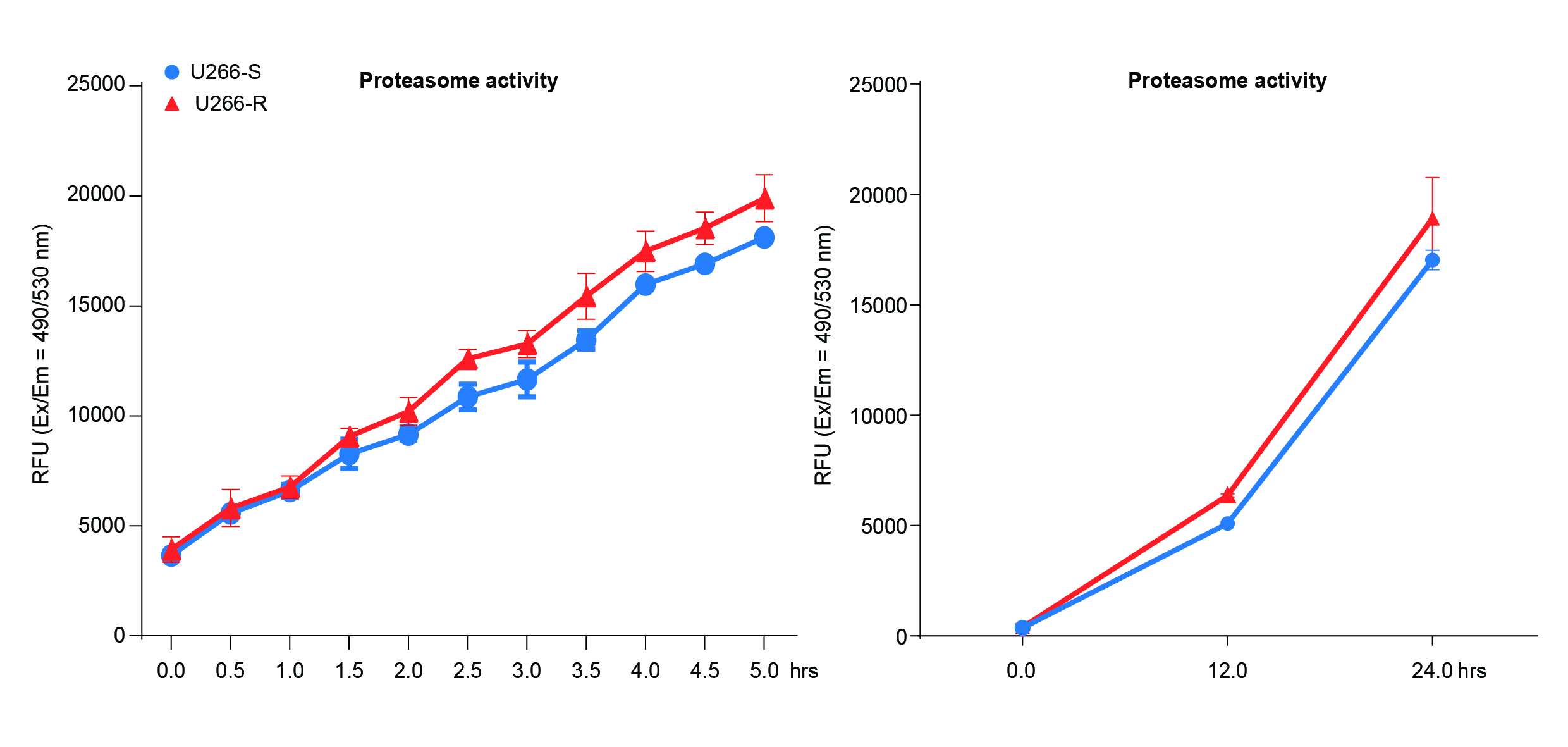

Supplement: Supplementary file 1 [file biomolecules-10-00696-s001.zip › Supplementary material/Figure_S1.jpg]
