# Supplementary material for: Mitochondrial Functions, Energy Metabolism and Protein Glycosylation are Interconnected Processes Mediating Resistance to Bortezomib in Multiple Myeloma Cells
Source: Biomolecules. 2020 Apr 30;10(5):696. doi: 10.3390/biom10050696 (PMC7277183; doi:10.3390/biom10050696)
Supplement: Supplementary file 1 [file biomolecules-10-00696-s001.zip › Supplementary material/Table S1.doc]

**Table S1.** Concentrations of GSH, as representative endogenous antioxidant, and nitrite and nitrate as stable end products of nitric oxide production and representative of nitrosative stress in U266-S and U266-R.

|  | **GSH** | **Nitrite** | **Nitrate** |
| --- | --- | --- | --- |
| **U266-S** | 1.10  (0.09) | 0.299  (0.121) | 0.289  (0.058) |
| **U266-R** | 1.65*  (0.34) | 0.051*  (0.014) | 0.224  (0.077) |

Values are the mean of four different experiments and are expressed as nmol/10^6^ cells. Standard deviations are in parenthesis. GSH=reduced glutathione.

^*^Significantly different from U266-S, p<0.05.
