# Supplementary material for: Mitochondrial Functions, Energy Metabolism and Protein Glycosylation are Interconnected Processes Mediating Resistance to Bortezomib in Multiple Myeloma Cells
Source: Biomolecules. 2020 Apr 30;10(5):696. doi: 10.3390/biom10050696 (PMC7277183; doi:10.3390/biom10050696)
Supplement: Supplementary file 1 [file biomolecules-10-00696-s001.zip › Supplementary material/Table S3.doc]

**Table S3. Concentrations of purine (GTP, GDP, GMP, IMP) and pyrimidine (UTP, UDP, UMP, CTP, CDP, CMP) nucleotides mono-, di-, and triphosphorylated in U266-S and U266-R.**

|  | **GTP** | **GDP** | **GMP** | **IMP** | **UTP** | **UDP** | **UMP** | **CTP** | **CDP** | **CMP** |
| --- | --- | --- | --- | --- | --- | --- | --- | --- | --- | --- |
| **U266-S** | 1.27  (0.15) | 0.206  (0.060) | 0.086  (0.032) | 0.204  (0.087) | 1.53  (0.18) | 0.344  (0.109) | 0.073  (0.013) | 0.455  (0.113) | 0.076  (0.017) | 0.012  (0.005) |
| **U266-R** | 1.60*  (0.19) | 0.176  (0.030) | 0.064  (0.018) | 0.115  (0.035) | 2.07*  (0.16) | 0.251  (0.058) | 0.038*  (0.013) | 0.729*  (0.104) | 0.052  (0.012) | 0.013  (0.007) |

Values are the mean of four different experiments and are expressed as nmol/10^6^ cells. Standard deviations are in parenthesis.

^*^Significantly different from U266-S, p<0.05.
