# Supplementary material for: Mitochondrial Functions, Energy Metabolism and Protein Glycosylation are Interconnected Processes Mediating Resistance to Bortezomib in Multiple Myeloma Cells
Source: Biomolecules. 2020 Apr 30;10(5):696. doi: 10.3390/biom10050696 (PMC7277183; doi:10.3390/biom10050696)
Supplement: Supplementary file 1 [file biomolecules-10-00696-s001.zip › Supplementary material/Table S2.doc]

**Table S2. Concentrations of adenine nucleotides in U266-S and U266-R.**

|  | **ATP** | **ADP** | **AMP** | **ECP** | **ATP/ADP** |
| --- | --- | --- | --- | --- | --- |
| **U266-S** | 3.91  (0.52) | 0.292  (0.052) | 0.103  (0.045) | 0.942  (0.12) | 14.10  (2.62) |
| **U266-R** | 4.50  (0.46) | 0.271  (0.035) | 0.071  (0.030) | 0.955  (0.010) | 16.62  (0.72) |

Values are the mean of four different experiments and are expressed as nmol/10^6^ cells. Standard deviations are in parenthesis. ECP = energy charge potential (ATP + 1/2ADP/ATP + ADP + AMP).
