# Supplementary material for: Mitochondrial Functions, Energy Metabolism and Protein Glycosylation are Interconnected Processes Mediating Resistance to Bortezomib in Multiple Myeloma Cells
Source: Biomolecules. 2020 Apr 30;10(5):696. doi: 10.3390/biom10050696 (PMC7277183; doi:10.3390/biom10050696)
Supplement: Supplementary file 1 [file biomolecules-10-00696-s001.zip › Supplementary material/Table S6.doc]

**Table S6.** Concentrations of AMP and ADP in U266-S and U266-R.

|  | **AMP** | **ADP** |
| --- | --- | --- |
| **U266-S** | 0.103  (0.045) | 0.282  (0.052) |
| **U266-R** | 0.071  (0.030) | 0.271  (0.035) |
|  |  |  |
| **U266-S+BTZ** | 0.934 ^#^  (0.186) | 0.997 ^#^  (0.130) |
| **U266-R+BTZ** | 0.238 ^*,#^  (0.056) | 0.668 ^*, #^  (0.137) |

Values are the mean of four different experiments and are expressed as nmol/10^6^ cells. Standard deviations are in parenthesis.

^*^Significantly different from U266-S or U266-S+BTZ, p<0.05.

^#^Significantly different from corresponding controls, p<0.05.
