# Supplementary material for: Mitochondrial Functions, Energy Metabolism and Protein Glycosylation are Interconnected Processes Mediating Resistance to Bortezomib in Multiple Myeloma Cells
Source: Biomolecules. 2020 Apr 30;10(5):696. doi: 10.3390/biom10050696 (PMC7277183; doi:10.3390/biom10050696)
Supplement: Supplementary file 1 [file biomolecules-10-00696-s001.zip › Supplementary material/Table S7.doc]

**Table S7.** Concentrations of purine (IMP, Hyp, Guanosine) and pyrimidine compounds (UMP, Uridine, Uracil, β-pseudouridine) in U266-S and U266-R.

|  | **IMP** | **Hyp** | **Guanosine** | **UMP** | **Uridine** | **Uracil** | **β-pseudouridine** |
| --- | --- | --- | --- | --- | --- | --- | --- |
| **U266-S** | 0.204  (0.087) | 0.033  (0.008) | 0.010  (0.006) | 0.073  (0.007) | 0.019  (0.002) | 0.359  (0.059) | 0.170  (0.021) |
| **U266-R** | 0.115  (0.035) | 0.027  (0.017) | 0.005  (0.003) | 0.038*  (0.013) | 0.014  (0.006) | 0.182*  (0.028) | 0.162  (0.018) |
|  |  |  |  |  |  |  |  |
| **U266-S+BTZ** | 0.917 ^#^  (0.300) | 0.196 ^#^  (0.059) | 0.079 ^#^  (0.027) | 0.392^#^  (0.087) | 0.488 ^#^  (0.064) | 0.419 ^#^  (0.044) | 0.495 ^#^  (0.128) |
| **U266-R+BTZ** | 0.319 ^*, b^  (0.082) | 0.050 ^*^  (0.016) | 0.027 *^, #^  (0.011) | 0.148 ^*, #^  (0.049 | 0.164 ^*, #^  (0.069) | 0.219 ^*^  (0.046)* | 0.221 ^*^  (0.050) |

Values are the mean of four different experiments and are expressed as nmol/10^6^ cells. Standard deviations are in parenthesis. IMP = inosine monophosphate; Hyp = hypoxanthine; UMP = uridine monophosphate.

^*^Significantly different from U266-S or U266-S+BTZ, p<0.05.

^#^Significantly different from corresponding controls, p<0.05.
