# Supplementary material for: Mitochondrial Functions, Energy Metabolism and Protein Glycosylation are Interconnected Processes Mediating Resistance to Bortezomib in Multiple Myeloma Cells
Source: Biomolecules. 2020 Apr 30;10(5):696. doi: 10.3390/biom10050696 (PMC7277183; doi:10.3390/biom10050696)
Supplement: Supplementary file 1 [file biomolecules-10-00696-s001.zip › Supplementary material/Table S5.doc]

**Table S5.** Concentrations of glycosylated UDP-derivatives under resting conditions in U266-S and U266-R.

|  | **UDP-Gal** | **UDP-Glc** | **UDP-GalNac** | **UDP-GlcNac** |
| --- | --- | --- | --- | --- |
| **U266-S** | 0.048  (0.010) | 0.281  (0.053) | 0.305  (0.058) | 0.641  (0.074) |
| **U266-R** | 0.086*  (0.012) | 0.391*  (0.045) | 0.460*  (0.078) | 1.16*  (0.084) |

Values are the mean of four different experiments and are expressed as nmol/10^6^ cells. Standard deviations are in parenthesis. UDP-Gal=UDP-galactose; UDP-Glc=UDP-glucose; UDP-GalNac=UDP-N-acetylgalactosamine; UDP-GlcNac=UDP-N-acetylglucosamine.

^*^Significantly different from U266-S, p<0.05.
