# Supplementary material for: Mitochondrial Functions, Energy Metabolism and Protein Glycosylation are Interconnected Processes Mediating Resistance to Bortezomib in Multiple Myeloma Cells
Source: Biomolecules. 2020 Apr 30;10(5):696. doi: 10.3390/biom10050696 (PMC7277183; doi:10.3390/biom10050696)
Supplement: Supplementary file 1 [file biomolecules-10-00696-s001.zip › Supplementary material/Table S4.doc]

**Table S4. Concentrations of oxidized and reduced nicotinic coenzymes under resting conditions in U266-S and U266-R.**

|  | **NAD^+^** | **NADH** | **NADP^+^** | **NADPH** | **NAD^+^/NADH** | **NADP^+^/NADPH** |
| --- | --- | --- | --- | --- | --- | --- |
| **U266-S** | 1.30  (0.14) | 0.158  (0.036) | 0.141  (0.008) | 0.068  (0.004) | 8.39  (1.44) | 2.07  (0.11) |
| **U266-R** | 1.51  (0.26) | 0.180  (0.034) | 0.148  (0.018) | 0.085*  (0.007) | 8.48  (0.76) | 1.75*  (0.21) |

Values are the mean of four different experiments and are expressed as nmol/10^6^ cells. Standard deviations are in parenthesis. NAD^+^=oxidized nicotinamide adenine dinucleotide; NADH = reduced nicotinamide adenine dinucleotide; NADP^+^=oxidized nicotinamide adenine dinucleotide phosphate; NADH = reduced nicotinamide adenine dinucleotide phosphate.

^*^ Significantly different from U266-S, p<0.05.
